# Proinflammatory Responses of 1-Nitropyrene against RAW264.7 Macrophages through Akt Phosphorylation and NF-κB Pathways

**DOI:** 10.3390/toxics9110276

**Published:** 2021-10-21

**Authors:** Ping-Kun Tsai, Shih-Pin Chen, Rosa Huang-Liu, Chun-Jung Chen, Wen-Ying Chen, Yan-Yan Ng, Yu-Hsiang Kuan

**Affiliations:** 1 Department of Internal Medicine, Zuoying Branch of Kaohsiung Armed Forces General Hospital, Kaohsiung 81342, Taiwan; pingkun@hotmail.com; 2 Department of Emergency Medicine, Tri-Service General Hospital, National Defense Medical Center, Taipei 11490, Taiwan; 3 Department of Internal Medicine, School of Medicine, Chung Shan Medical University, Taichung 40201, Taiwan; cshy752@csh.org.tw; 4 Department of Internal Medicine, Chung Shan Medical University Hospital, Taichung 40201, Taiwan; 5 School of Nutrition, Chung Shan Medical University, Taichung 40201, Taiwan; rhl@csmu.edu.tw; 6 Department of Education and Research, Taichung Veterans General Hospital, Taichung 40705, Taiwan; cjchen@vghtc.gov.tw; 7 Department of Veterinary Medicine, National Chung Hsing University, Taichung 40227, Taiwan; wychen@dragon.nchu.edu.tw; 8 Department of Pediatric, Chung Kang Branch, Cheng Ching Hospital, Taichung 40764, Taiwan; angeleng77@gmail.com; 9 Department of Pharmacy, Chung Shan Medical University Hospital, Taichung 40201, Taiwan; 10 Department of Pharmacology, School of Medicine, Chung Shan Medical University, Taichung 40201, Taiwan

**Keywords:** 1-nitropyrene, proinflammatory cytokines, PGE2, NF-κB pathway, Akt

## Abstract

Air pollution is a major environmental and public health problem worldwide. A nitro-polycyclic aromatic hydrocarbon and the most abundant air pollutant in diesel engine exhaust, 1-nitropyrene (1-NP), is caused by the incomplete combustion of carbonaceous organic substances. Macrophages are effector cells of the innate immune cells that provide resistance in the peripheral tissue. The overactivation of macrophages results in inflammation. The generation of proinflammatory cytokines, such as interleukin (IL)-1β, IL-6, and tumour necrosis factor alpha, is induced by 1-NP in a concentration-dependent manner in macrophages. In this study, the production of proinflammatory mediators, such as nitrogen oxide and prostaglandin E2, was induced by 1-NP in a concentration-dependent manner through the expression of iNOS and COX2. The generation of proinflammatory cytokines, iNOS, and COX2 was induced by 1-NP through nuclear factor (NF)-κB p65 phosphorylation and the degradation of its upstream factor, IκB. Finally, Akt phosphorylation was induced by 1-NP in a concentration-dependent manner. These findings suggest that 1-NP exhibits a proinflammatory response through the NF-κB pathway activation due to Akt phosphorylation.

## 1. Introduction

Air pollution is caused by a mixture of pollutants floating in the atmosphere. These pollutants are generated from natural events, such as fires, storms, volcanic eruptions, and the release of pollen. Furthermore, a major source of air pollutants is human activity, such as fossil fuel combustion, agricultural activity, manufacturing (particularly in the form of factory exhausts), and mining [1,2,3]. Exposure to air pollutants is associated with many diseases, including cardiovascular diseases, neurodegenerative diseases, diabetes, and glaucoma [1,2,4,5]. Known as a nitro-polycyclic aromatic hydrocarbon and the most abundant nitroarene in diesel engine exhaust, 1-nitropyrene (1-NP) results from the incomplete combustion of carbonaceous organic substances [6,7]. Genotoxicity and apoptosis are induced by 1-NP in macrophages, liver and lung epithelial cells, and hepatoma cells [8,9,10,11,12]. Chronic inflammation in pulmonary fibrosis is induced by 1-NP through endoplasmic reticulum stress in mice [13]. Cytokines and chemokines are induced by 1-NP in the alveolar epithelial cell line A549 and the human bronchial epithelial cell line BEAS-2B [14,15].

Inflammation is a crucial defence mechanism against invasive microorganisms and inhaled air pollution, including diesel engine exhaust and particulate matter. The elimination of microorganisms and air pollutants due to proinflammatory mediators is accompanied by inflammation in peripheral tissues [16,17,18]. Macrophages are effector cells of the innate immune cells that provide resistance in the peripheral tissue [16]. Therefore, macrophage activation is involved in the action of proinflammatory mediators, including cytokines, nitrogen oxide (NO), and prostaglandin E2 (PGE2) [19,20]. The activation of the proinflammatory transcript factor nuclear factor (NF)-κB is induced by environmental pollutants in macrophages through the phosphoinositide 3-kinase (PI3K) and Akt signalling pathways [21,22]. However, the effects of 1-NP-induced inflammation in macrophages remain unclear. Thus, this study examined the proinflammatory responses and relative mechanisms of macrophage RAW264.7 cells treated with 1-NP.

## 2. Materials and Methods

### 2.1. Materials

Primary antibodies for cyclooxygenase-2 (COX-2), inducible nitric oxide synthase (iNOS), phosphoryl (P)-Akt, Akt, P-p38 mitogen-activated protein kinase (MAPK), p38 MAPK, P-p65, p65, IκB, and β-actin were obtained from Santa Cruz Biotechnology (Santa Cruz, CA, USA). All secondary antibodies used for Western blot assays were purchased from Jackson ImmunoResearch Laboratories (Baltimore, MD, USA). ELISA kits for interleukin (IL)-1β, IL-6, tumour necrosis factor (TNF) α, and prostaglandin (PG) E2 were purchased from Cayman Chemicals (Ann Arbor, MI, USA). All agents for cell culture were purchased from HyClone Laboratories (Logan, UT, USA). A Griess reaction assay kit, 1-NP, phosphate-buffered saline (PBS), dimethyl sulphoxide (DMSO), and other agents were obtained from Sigma-Aldrich (St. Louis, MO, USA). In this work, 1-NP was dissolved in DMSO and the final concentration of DMSO was found to be less than 0.05% in each test sample.

### 2.2. Cell Culture and Treatment

RAW264.7 murine macrophage-like cells purchased from the Bioresource Collection and Research Centre (BCRC, Hsinchu, Taiwan) were cultured in DMEM supplemented with 50 IU/mL penicillin and 100 µg/mL streptomycin at 37 °C in an atmosphere of 5% CO_2_ and 95% air. RAW264.7 cells were plated at a density of 5 × 10^5^ cells/mL for 12 h, then the medium was replaced with a serum-free DMEM medium [11,12]. After cell incubation with 1-NP at the concentrations of 0, 3, 10, 30, and 50 μM for 30 min or 12 h, supernatants were collected for an enzyme-linked immunosorbent assay (ELISA) and Griess reaction assays; the cells were harvested for a protein expression assay.

### 2.3. ELISA Assay

The levels of interleukin (IL)-1β, IL-6, tumour necrosis factor alpha (TNFα), and PGE2 were measured using an ELISA assay kit according to the manufacturer’s instructions and the assay procedures of a previous study [23,24]. After cells were incubated with 1-NP at the indicated concentrations for 12 h, the supernatants were harvested and loaded into plates coated with anticytokine antibodies for 16 h at 4 °C. After being washed, biotin-conjugated anticytokine antibodies were placed onto a plate for 2 h at room temperature. After washing, streptavidin HRP solution and TMB substrate solution were loaded onto the plate. Sulfuric acid was used to stop the reaction and the absorbance was measured at 450 nm using a microplate reader (Synergy HT Multi-Mode Microplate Reader, Biotek, Winooski, VT, USA).

### 2.4. Griess Reaction Assay

After the cells were incubated with 1-NP at the indicated concentrations for 12 h, the level of NO production in the medium was detected with the Griess reaction. The same volumes of 1% sulphanilamide dissolved in 5% phosphoric acid and 0.1% N-(1-naphthyl) ethylenediamine dihydrochloride dissolved in water were added to the medium. The absorbance at 540 nm was determined using a microplate reader. The nitrite concentration in the samples was determined with reference to the dilution of sodium nitrite as a standard [20].

### 2.5. Western Blotting Assay

After cells were incubated with 1-NP at the indicated concentrations for 12 h, they were washed with ice-cold PBS and solubilised in RIPA lysis buffer [23,24]. Equal amounts of the protein were denatured in SDS, electrophoresed on SDS–PAGE, and transferred to polyvinylidene difluoride membranes. The membranes were probed with primary antibodies. After being washed, the membranes were re-probed with HRP-conjugated secondary antibodies. All membranes were developed and quantified with enhanced chemiluminescence labelling solution using an Infinity CX5 detection system (Vilber Lourmat, France).

### 2.6. Statistical Analysis

All the experiments were repeated at least thrice. Values were expressed in terms of the mean ± standard deviation (S.D.). The significance of the difference from the respective controls for each experimental test condition was assessed using a one-way ANOVA, followed by Bonferroni’s multiple comparisons post hoc test. Data were analysed using the SPSS software. A *p* value <0.05 was considered significant.

## 3. Results

### 3.1. Effects of 1-NP on Proinflammatory Cytokine Generation in RAW264.7 Macrophages

**Figure 1:**
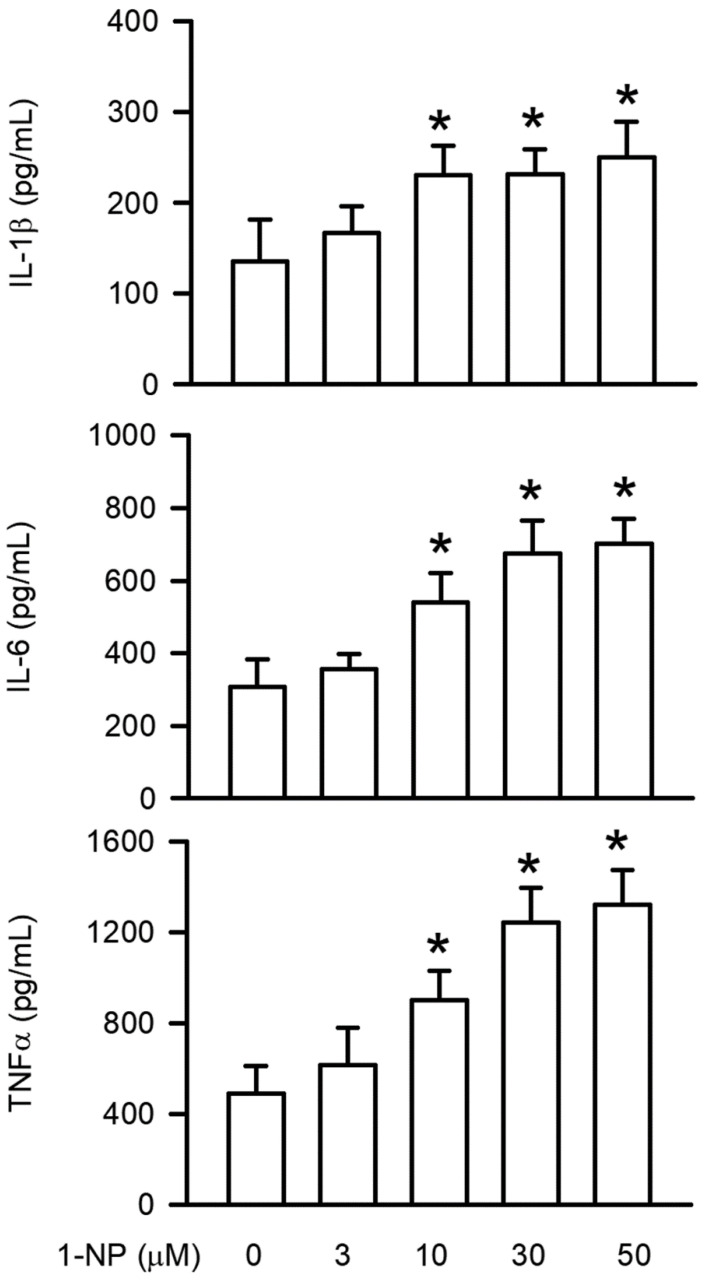
Proinflammatory cytokine generation was induced by 1-NP in RAW264.7 macrophages. Proinflammatory cytokines, including IL-1β, IL-6, and TNFα, were determined by the ELISA assay. Results are expressed as means ± SD (*n* = 4). * *p* < 0.05 is considered significant, compared with the control group, which indicated treatment with 1-NP at 0 μM.

ELISA assays were used to quantify proinflammatory cytokines, such as IL-1β, IL-6, and TNFα, generated in the medium of RAW264.7 macrophages. As illustrated in Figure 1, 1-NP-treated cells produced significantly more proinflammatory cytokines than the control cells (*p* < 0.05). Proinflammatory cytokine inhibition presented in a concentration-dependent manner and a significant increase was seen starting at 10 μM (*p* < 0.05).

### 3.2. Effects of 1-NP on NO Generation and iNOS Expression in RAW264.7 Macrophages

**Figure 2:**
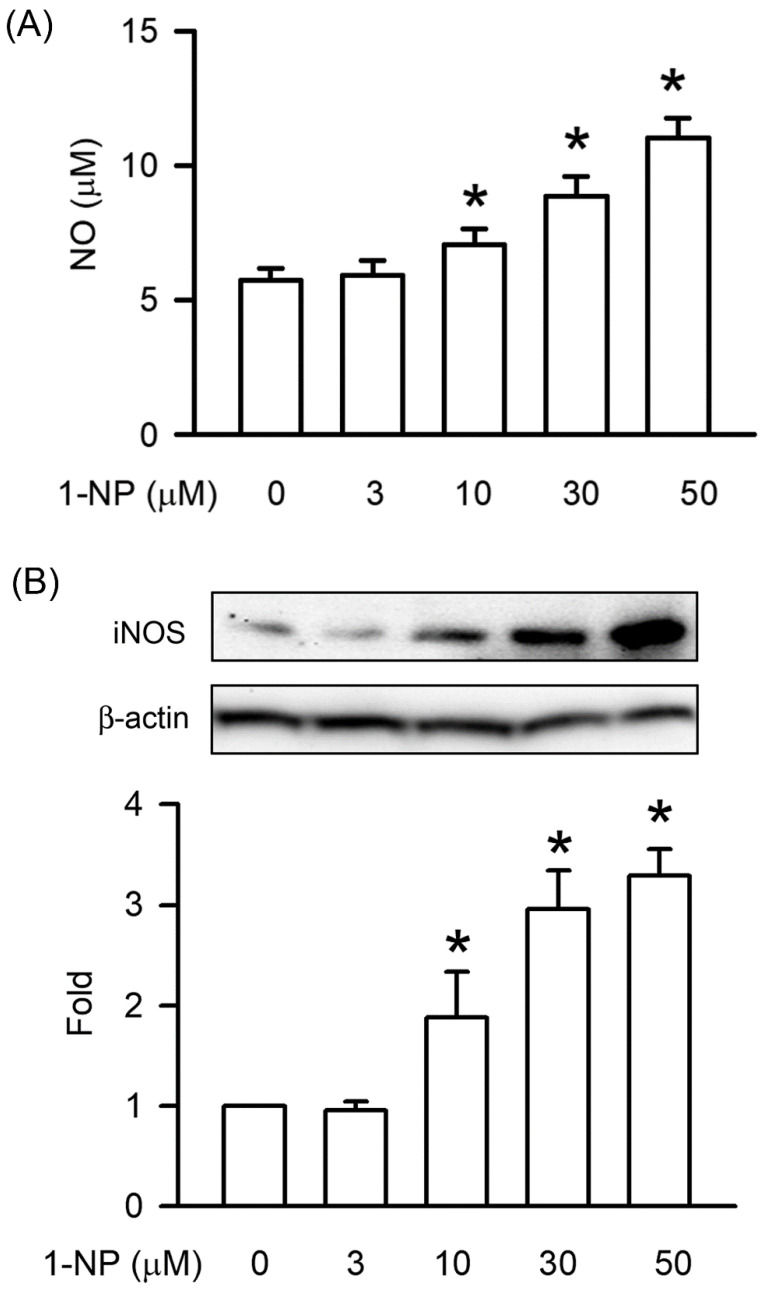
NO generation and iNOS expression were induced by 1-NP in RAW264.7 macrophages. The level of NO generation (**A**) and iNOS expression (**B**) was measured by a Griess reaction assay and Western blotting assay, respectively. Results are expressed as means ± SD (*n* = 3). * *p* < 0.05 is considered significant, compared with the control group, which indicated treatment with 1-NP at 0 μM.

The NO generation induced by 1-NP was measured using a Griess reaction assay on RAW264.7 macrophages. After RAW264.7 macrophages had been incubated with 1-NP for 12 h, PGE2 and NO were generated in a concentration-dependent manner and a significant increase was observed at 10 μM (*p* < 0.05, Figure 2). iNOS is the upstream factor of NO generation. The expression of iNOS was induced by 1-NP in a concentration-dependent manner, and a significant increase was seen starting at 10 μM (*p* < 0.05, Figure 2).

### 3.3. Effects of 1-NP on PGE2 Production, COX2 Expression, and cPLA2 Phosphorylation in RAW264.7 Macrophages

**Figure 3:**
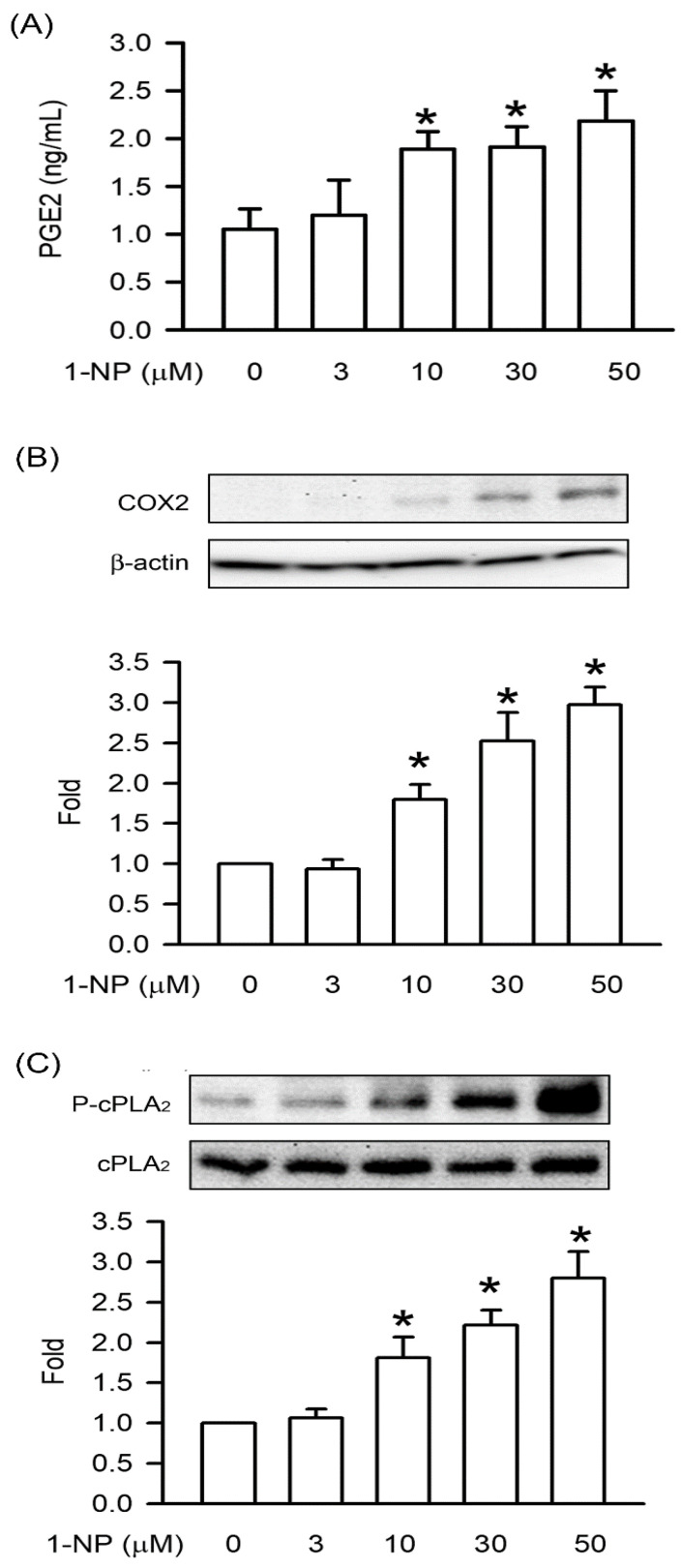
PGE2 production, COX2 expression, and cPLA2 phosphorylation were induced by 1-NP in RAW264.7 macrophages. (**A**) The level of PGE2 generation was measured by ELISA assay. The level of COX2 expression (**B**) and cPLA2 phosphorylation (**C**) was measured by Western blotting assay. Results are expressed as means ± SD (*n* = 3). * *p* < 0.05 is considered significant, compared with the control group, which indicated treatment with 1-NP at 0 μM.

The PGE2 production induced by 1-NP was measured using an ELISA assay. After RAW264.7 macrophages were incubated with 1-NP for 12 h, PGE2 production was induced by 1-NP in a concentration-dependent manner, and a significant increase was seen starting at 10 μM (*p* < 0.05, Figure 3). COX2 expression and cPLA2 phosphorylation are upstream factors of PGE2 production. COX2 expression and cPLA2 phosphorylation were induced by 1-NP in a concentration-dependent manner, and a significant increase was seen starting at 10 μM (*p* < 0.05, Figure 3).

### 3.4. Effects of 1-NP on IκB Degradation and NFκB p65 Phosphorylation in RAW264.7 Macrophages

**Figure 4:**
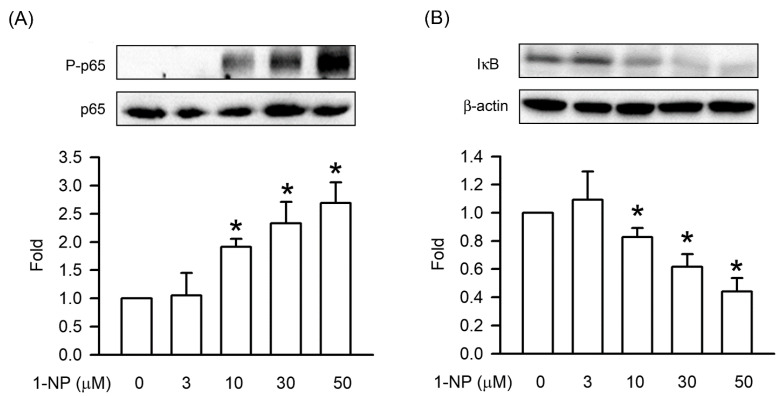
NFκB p65 phosphorylation and IκB degradation were induced by 1-NP in RAW264.7 macrophages. The level of NFκB p65 phosphorylation (**A**) and IκB degradation (**B**) was measured by a Western blotting assay. Results are expressed as means ± SD (*n* = 3). * *p* < 0.05 is considered significant, compared with the control group, which indicated treatment with 1-NP at 0 μM.

The activation of the proinflammatory transcript factor NF-κB p65 plays an essential role in the generation of proinflammatory cytokines and mediators. IκB degradation and NFκB p65 phosphorylation play important roles in NF-κB activation. IκB degradation and NFκB p65 phosphorylation were significantly higher relative to such levels in control cells after treatment with 1-NP for 12 h (*p* < 0.05, Figure 4). The phosphorylation of p65 presented in a concentration-dependent manner, and a significant increase was seen starting at 10 μM (*p* < 0.05, Figure 4).

### 3.5. Effects of 1-NP on Akt Phosphorylation in RAW264.7 Macrophages

**Figure 5:**
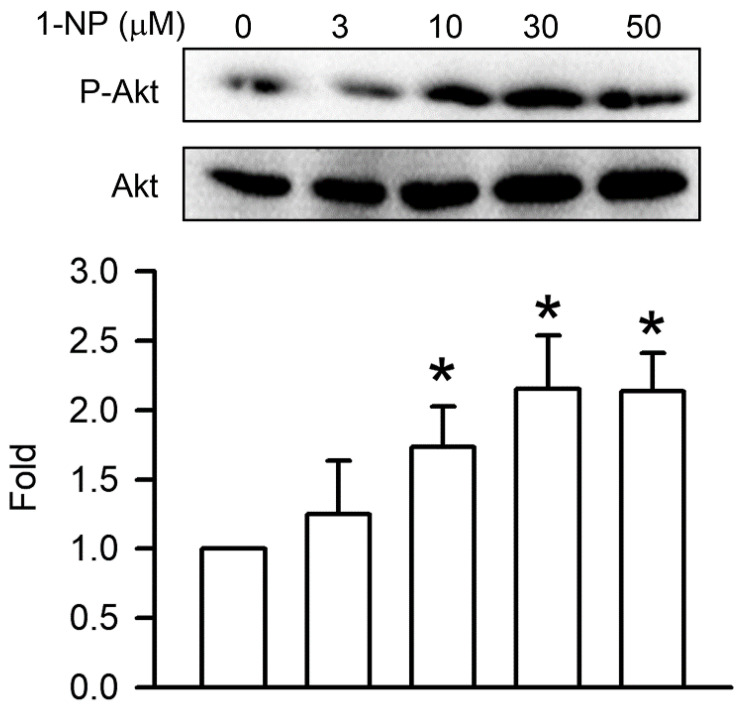
Akt phosphorylation was induced by 1-NP in RAW264.7 macrophages. The level of Akt phosphorylation was measured by a Western blotting assay. Results are expressed as means ± SD (*n* = 3). * *p* < 0.05 is considered significant, compared with the control group, which indicated treatment with 1-NP at 0 μM.

Akt phosphorylation is a crucial upstream factor in NF-κB activation and involves IκB degradation. Akt phosphorylation was analysed in cells treated with 1-NP for 30 min. The phosphorylation of Akt in RAW264.7 cells stimulated with 1-NP increased markedly in a concentration-dependent manner, and a significant increase was seen starting at 10 μM (*p* < 0.05, Figure 5).

## 4. Discussion

The solubility and concentration of 1-NP are very low in the gas phase [25]. However, 1-NP is adsorbed as an air pollutant from diesel engine exhaust fumes and particulate matter. Previous studies have reported that concentrations of 1-NP from 0.5 to 275 μM in exhaust emissions from gasoline-powered engines, light-duty diesel-powered engines, and heavy-duty diesel-powered engines [26]. Therefore, we employed 1-NP at a level of 3 to 50 μM for this study. In our previous studies, we found that cytotoxicity was induced by 1-NP in a time- and concentration-dependent manner. After 1-NP was incubated with RAW264.7 cells for 12 h, the cytotoxicity was approximately 10, 20, and 35% at concentrations of 10, 30, and 50 μM, respectively. After 1-NP was incubated with RAW264.7 cells for 24 h and 48 h, more serious levels of cytotoxicity were generated [11,12]. Based on these findings, we suggest that cytotoxicity induced by 1-NP cannot be recovered. The longer it takes to treat cells with 1-NP, the greater are the harmful effects on the cells, and the more likely it is that the caused damage leads to cytotoxicity.

In previous studies, we found that cytotoxicity was induced by 1-NP in a time- and concentration-dependent manner. With the same duration of 1-NP treatment of 12 h, the level of cytotoxicity was approximately 10, 20, and 35% at concentrations of 10, 30, and 50 mM, respectively. After 1-NP was incubated with RAW264.7 cells for 24 and 48 h, more serious levels of cytotoxicity were generated. These findings suggest that the cytotoxicity induced by 1-NP cannot be recovered. In addition, the cytotoxicity and genotoxicity induced by 1-NP may occur via intracellular accumulation and metabolism [11,12]. Based on these findings, we hypothesise that the proinflammatory response is higher than the toxic effects after 1-NP is incubated with RAW264.7 cells for 12 h. 

Macrophages play a crucial role in the innate immunity system in phagocytosis, digestion, and the elimination of invasive pathogens, microorganisms, and air pollutants through the generation of proinflammatory cytokines such as IL-1β, IL-6, and TNFα [27,28,29]. In addition to the linkage between innate and adaptive immunity, IL-1β, IL6, and TNFα were found to induce acute and strong inflammatory responses, including leukocyte recruitment and proinflammatory mediator generation. These inflammatory responses cause excessive damage to peripheral tissues and then result in pathological diseases, such as atherosclerosis, adult respiratory distress syndrome, and tumours [30,31,32]. The levels of IL-1β, IL6, and TNFα in mRNA are upregulated by 1-NP in mouse lungs [33]. The generation of IL-1β, IL6, and TNFα is induced by 1-NP in the human alveolar basal epithelial cell line, specifically in A549 cells, and the human bronchial epithelial cell line, specifically in BEAS-2B cells [15,29]. The findings of this study revealed that the production of IL-1β, IL6, and TNFα is induced by 1-NP in a concentration-dependent manner in RAW264.7 cells. These results suggest that proinflammatory cytokines are induced by 1-NP in macrophages.

In inflammatory pathogenesis, the excessive production of NO and PGE2 is an important contributor. After macrophages were treated with proinflammatory stimuli, NO and PGE2 were generated from iNOS and COX-2, respectively. In addition, cPLA2 phosphorylation was involved in COX-2-dependent PGE2 generation in macrophages [19,34]. This result is the first to demonstrate that 1-NP-induced NO generation occurs through iNOS upregulation and PGE2 production by the upregulation of COX-2 through cPLA2 phosphorylation. The present results reveal that inflammatory responses are induced by 1-NP in macrophages through the generation of proinflammatory mediators, such as NO and PGE2, and proinflammatory cytokines, such as IL-1β, IL6, and TNFα.

NF-κB acts as the major nuclear transcription factor promoter and stimulates the generation of proinflammatory mediators. NF-κB p65, also called RelA, is the most abundant functional subunit of NF-κB. IκB is a critical upstream factor of NF-κB activation. In the normal resting state of macrophages, IκB binds with NF-κB to create complex resistance in the cytosol [24,35]. After macrophages are exposed to pollutants and stimulants, NF-κB p65 is liberated from the IκB-NF-κB complex, translocated from the cytosol to nuclear, and bound to DNA to enhance the expression of proinflammatory mediators. At the same time, IκB is degraded by the proteasome in the cytosol. As has been shown previously, the phosphorylation of NF-κB p65 was increased by 1-NP in A549 cells and mouse lungs [33]. In this study, we further found the phosphorylation of p65 through the degradation of IκB in 1-NP-treated RAW264.7 macrophages. These results suggest that the generation of proinflammatory mediators is stimulated by 1-NP via the activation of the NF-κB pathway, including NF-κB p65 phosphorylation and IκB degradation.

In the pathogenesis of inflammation, the PI3K/Akt pathway plays a key role in mammal cells and animals. Akt, also named protein kinase B, is an important downstream effector of PI3K [36]. The activation of the NF-κB pathway is enhanced by Akt phosphorylation in the macrophages. After the treatment of mouse lungs and A549 cells with 1-NP, the phosphorylation of Akt was upregulated [10,33]. Here, the phosphorylation of Akt was induced by 1-NP in macrophages. Moreover, the concentration trend of Akt phosphorylation is similar to that of NF-κB pathway activation, including NF-κB p65 phosphorylation and IκB degradation, in 1-NP treated with RAW264.7 macrophages. These results indicate that NF-κB pathway activation is induced by 1-NP through Akt phosphorylation.

Our results demonstrate that the generation of proinflammatory mediators, such as NO and PGE2, is induced by 1-NP through the expression of iNOS and COX2. The expression of proinflammatory cytokines, including IL-1β, IL-6, and TNFα, was also induced by 1-NP. The expression of proinflammatory mediators and cytokines was induced by 1-NP through the activation of the NF-κB pathway and its upstream factor, Akt phosphorylation. In conclusion, inflammatory responses were induced by 1-NP through the activation of the NF-κB pathway via Akt phosphorylation. 

## Data Availability

The data presented in this study are available on request from the corresponding author.
